# A novel knee implant for total knee arthroplasty meets expectations at 10 years. First long‐term follow‐up report of clinical outcomes and survivorship

**DOI:** 10.1002/ksa.70037

**Published:** 2025-08-29

**Authors:** Alice Montagna, Marina Marescalchi, Virginia Cinelli, Rudy Sangaletti, Luca Andriollo, Francesco Benazzo, Stefano Marco Paolo Rossi

**Affiliations:** ^1^ U.O.C Ortopedia e Traumatologia Fondazione Poliambulanza Brescia Italy; ^2^ Clinica Ortopedica e Traumatologica Fondazione IRCCS Policlinico San Matteo Pavia Italy; ^3^ Università Cattolica del Sacro Cuore Rome Italy; ^4^ IUSS Istituto Universitario di Studi Superiori Pavia Italy; ^5^ Unità di Chirurgia Robotica U.O.C Ortopedia e Traumatologia, Fondazione Poliambulanza Brescia Italy; ^6^ Department of Life Science Health, and Health Professions Università degli Studi “Link Campus University” Roma Italy

**Keywords:** J‐curve, new designs, novel implant, posterior stabilised, total knee replacement

## Abstract

**Purpose:**

Achieving a “forgotten knee” after total knee arthroplasty (TKA) remains a primary goal in modern knee replacement surgery. Anatomic implant designs aim to replicate native knee anatomy and kinematics, potentially improving patient satisfaction and functional outcomes. This study evaluates the long‐term clinical outcome and survivorship of the Persona Knee System at a minimum follow‐up of 10 years.

**Methods:**

116 TKAs performed using the Persona Posterior Stabilised (PS) Knee System (Zimmer Biomet, Warsaw, Indiana, USA) between 2013 and 2014 at a high‐volume orthopaedic centre were prospectively followed and retrospectively analysed. Clinical outcomes were assessed using patient‐reported outcome measures (PROMs), including the Forgotten Joint Score‐12 (FJS‐12), Oxford Knee Score (OKS), and Western Ontario and McMaster Universities Arthritis Index (WOMAC). Implant survivorship was determined using Kaplan‐Meier analysis, and complication rates were recorded.

**Results:**

At a mean follow‐up of 11.1 years, 116 knees were available for analysis. Patients were assessed clinically and radiographically at 1, 3 and 6 months postoperatively, and then annually, with a mean follow‐up of 11.1 years. The mean FJS‐12 was 69.52 (SD 12.21, range 15–88), indicating a high level of joint awareness reduction. The OKS and WOMAC scores significantly improved postoperatively, with mean final values of 38.63 (SD 7.99, range 7–48) and 25.29 (SD 16.97, range 4–91), respectively. Radiological analysis demonstrated accurate and stable implant positioning, with no progressive radiolucent lines in non‐revised cases. Kaplan–Meier survival analysis showed a 95.7% (SD 1.9%) implant survival rate. The revision rate was 4.3%, with aseptic loosening and persistent painful prosthesis as the primary causes.

**Conclusion:**

The anatomic design of the Persona Knee System provides excellent long‐term clinical outcomes, high patient satisfaction, and sustained implant durability. Future research should further investigate patient‐specific factors and surgical refinements to optimise long‐term outcomes in TKA.

**Level of Evidence:**

Level III.

**Abbreviations**
FJS‐12: Forgotten Joint Score‐12
OKS: Oxford Knee Score
PCL: posterior cruciate ligament
PS: posterior stabilised
TKA: total knee arthroplasty
VTE: venous thromboembolism
WOMAC: Western Ontario and McMaster Universities Arthritis Index

## INTRODUCTION

Knee osteoarthritis (OA) affects over 368 million people globally, with cases projected to rise by 74% by 2050 [1, 5, 11, 28]. While Total knee arthroplasty (TKA) is the most effective surgical treatment for advanced OA, achieving very satisfactory clinical and survival outcomes, research on design and surgical technique is focusing on improving patient's satisfaction and knee kinematics [6, 10, 12, 14, 29].

Recent studies have highlighted several factors that positively influence patient outcomes in TKA moving closer to the ultimate goal of delivering a “forgotten knee” experience for patients [2, 17, 19, 25, 32, 34]. These include refined surgical techniques that minimise soft tissue trauma; precise component alignment, which plays a critical role in ensuring proper joint function and reducing wear over time; advancements in prosthesis design, which aim to mimic the natural biomechanics of the knee [21, 27]. Established systems have shown durable results but may lack the anatomical precision needed to optimise patient satisfaction.

The Persona Knee System, (Zimmer Biomet, Warsaw, Indiana, USA), which is now on the market since 10 years, was developed to replicate native knee anatomy and kinematics more closely than previous generations. It features anatomically shaped femoral and tibial components, offered in a wide range of sizes and combinations, enabling a personalised fit that minimises issues such as overhang or undercoverage. The femoral component has a J‐Curve design and includes a contoured trochlear groove designed to mimic the native anatomy, optimising patellar tracking and reducing anterior knee pain. The tibial baseplate enhances rotational alignment and cortical bone coverage, supporting improved load distribution and implant stability. Asymmetric polyethylene inserts further aim to replicate joint line obliquity, contributing to the restoration of physiological kinematics and potentially improving long‐term outcomes and patient satisfaction.

The aim of this study is to evaluate the clinical and radiological outcomes, as well as long‐term survival rate of this anatomic total knee replacement design,. By analysing patient satisfaction, functional recovery, and implant durability over an extended follow‐up period, this research, at present time the first conducted on long term data, seeks to determine whether the advanced design and personalised approach of the Persona Knee System provides sustained, natural joint function, reduced pain, and high durability at long‐term follow‐up.

## MATERIALS AND METHODS

This study prospectively followed and retrospectively evaluated patients who underwent total knee arthroplasty using the Persona Knee System (Zimmer Biomet, Warsaw, Indiana, USA) between January 2013 and December 2014 at a high‐volume orthopaedic centre. All procedures were performed by two experienced knee arthroplasty surgeons (senior authors).

Between January 2013 and December 2014, a total of 149 knees in 137 patients who underwent TKA using the Persona implant system were included in the study.

At the time of final evaluation, 22 patients (including one with bilateral TKAs) had passed away, and nine patients (including one with bilateral TKAs) were lost to follow‐up. A total of 10 patients underwent bilateral procedures, resulting in 116 knees analysed from 106 patients.

**Figure 1:**
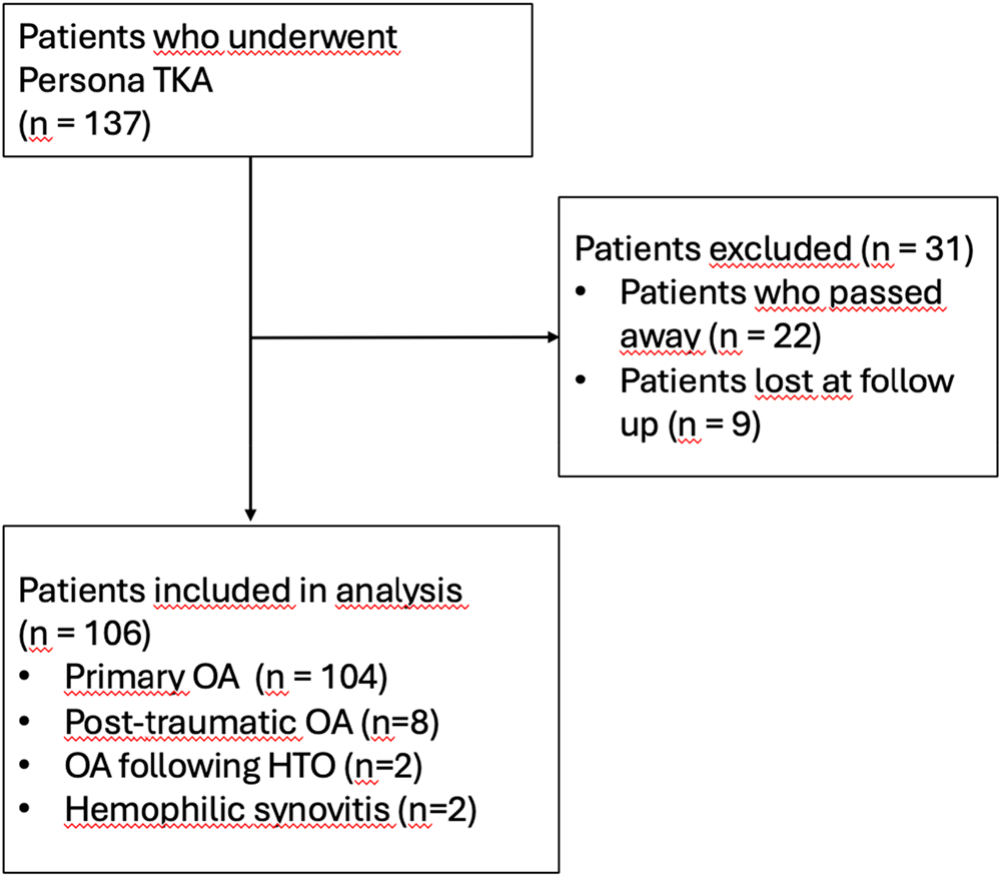
Participants flow diagram. HTO, high tibial osteotomy; OA, osteoarthritis; TKA, total knee arthroplasty.

The primary indications for TKA were as follows: 104 cases of primary osteoarthritis, eight cases of post‐traumatic arthritis, two cases of ostheoartritis following medial‐wedge high tibial osteotomy (HTO) and two cases of haemophilic synovitis (Figure 1).

The final cohort consisted of 62% females and 38% males, with a mean age at surgery of 65.3 ± 10.7 years. Among the 116 knees, 44% were left‐sided and 56% were right‐sided.

The mean BMI of the cohort was 27.39 ± 2.84 kg/m^2^, with an average height of 165.35 ± 9.21 cm and an average weight of 78.79 ± 12.11 kg. Patients' demographics at time of surgery are summarised in Table 1.

**Table 1:** Patients demographics.

Patient population	Number	%
Total knees	116	100%
Total patients	106	100%
Male	44	38%
Female	72	62%
Left knee	51	44%
Right knee	65	56%
Indication		
Primary OA	104	89.65%
Post‐traumatic OA	8	6.89%
Haemophilic synovitis	2	1.72%
Post high tibial osteotomy	2	1.72%
Type of anaesthesia		
General	8	6.90%
Spinal	108	93.10%
	Average	S.D.
Age at surgery (years)	65.3	10.7
BMI	27.39	2.84
Height (cm)	165.35	9.21
Weight (kg)	78.79	12.11
FU (months)	133	7.54
Surgical time		
Single procedures (min)	98.61	18.28
Bilateral procedures (min)	202	29.28

Abbreviations: BMI, body mass index; OA, osteoarthritis.

The patella was resurfaced in 113 out of 116 cases (97.4%). Patients were eligible for inclusion if they required TKA due to primary osteoarthritis, inflammatory arthritis such as rheumatoid arthritis, avascular necrosis (AVN), or post‐traumatic arthritis. Additional inclusion criteria required a minimum follow‐up period of 10 years and the availability of complete clinical and radiological data.

Exclusion criteria included a history of active or recent joint infections such as septic arthritis, significant bone defects or severe deformities that necessitated the use of more highly constrained prostheses (such as hinged or constrained implants), neuromuscular disorders that impaired joint stability or gait (e.g., Parkinson's disease or residual hemiparesis from stroke) and known allergies or hypersensitivity to the materials used in the Persona Knee System.

### Surgical technique

All surgeries were performed under either general or spinal anaesthesia, based on the anaesthesiologist's evaluation and patient‐specific considerations. The operations were conducted using a medial parapatellar approach and a manual measured resection technique, aiming to achieve neutral mechanical alignment in the coronal plane. Both tibial and femoral alignment was guided by extramedullary referencing. After performing the distal femoral and proximal tibial cuts, the flexion space was created using a measured resection technique. Femoral sizing was carried out using anterior referencing, with rotational alignment determined based on standard bony landmarks.

The rotational alignment could be further refined by the surgeon using the anterior‐posterior (AP) sizer, allowing for up to 4° of correction in either direction, or with the additional shift block, enabling up to 2° of further adjustment. The initial evaluation of rotational alignment was conducted with paddles positioned flush against the posterior condyles, influenced by ligament tension and the posterior condylar line. A secondary evaluation, independent of the posterior cruciate ligament (PCL), was performed with the 4‐in‐1 cutting jig to verify true rotational alignment and ensure equal flexion and extension spaces.

In every case, the cemented posterior‐stabilized (PS) version of the Persona knee system was implanted, and 97.4% patellar resurfacing was performed. In all cases, an antibiotic‐impregnated cement containing gentamicin and vancomycin was used.

### Postoperative protocol and rehabilitation

All patients received venous thromboembolism (VTE) prophylaxis with low‐molecular‐weight heparin for the first 30 days following surgery, in addition to the use of elastic compression stockings.

Patients were encouraged to begin immediate full weight‐bearing with crutches for the first 30 days. There were no imposed movement restrictions, and physical therapy focused on achieving active flexion and extension early in the postoperative period.

### Outcome measures and data collection

Patients were assessed clinically and radiographically at 1, 3, 6 months and then yearly. Patient‐reported outcome measures were utilised to comprehensively evaluate the clinical outcomes at last follow up. The WOMAC was used to assess three key domains: pain, stiffness, and physical function [23].

The OKS was employed as a knee‐specific measure to evaluate pain and functional limitations in daily activities, such as walking, climbing stairs and rising from a seated position [33].

Finally, Forgotten Joint Score‐12 (FJS‐12) was included to specifically assess whether patients achieved the “forgotten knee,” a state in which the knee replacement becomes so natural and integrated into daily activities that it is no longer consciously perceived [31].

For patients who required revision surgery, detailed data on the reasons for revision and specific procedures performed were collected. Survival rates of the Persona Knee System were calculated based on implant longevity and the absence of revision procedures (Kaplan Meyer analysis).

### Radiographic analysis

**Figure 2:**
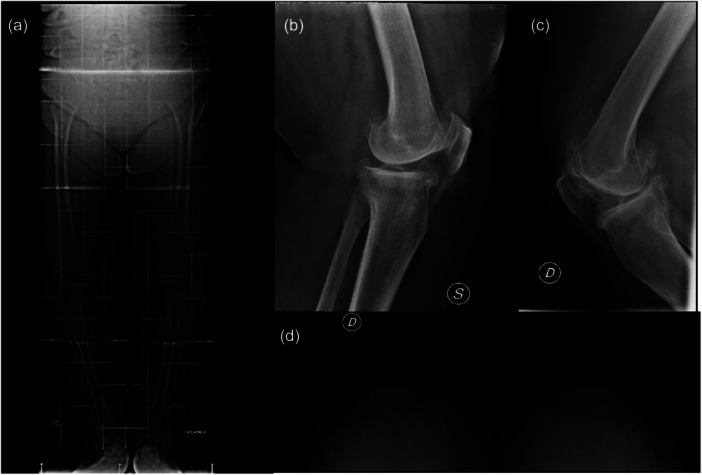
Preoperative full‐length standing radiograph of the lower limbs (a), lateral (b, c) and axial patellar (d) views of a bilateral case.

Preoperative imaging included anteroposterior, lateral, and axial patellar radiographs, along with a full‐length standing radiograph of the lower limbs to assess alignment (Figure 2a–d).

**Figure 3:**
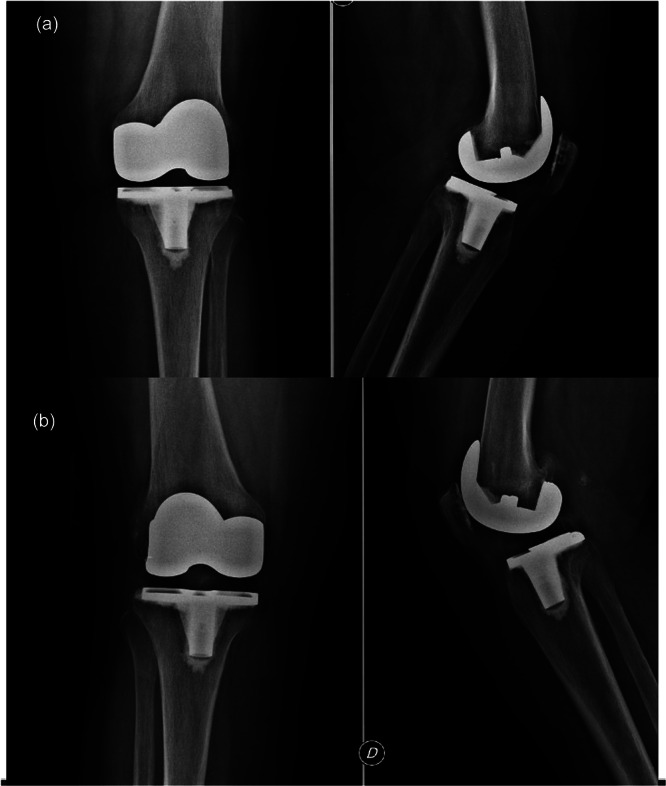
Post operative X rays of the left knee (a) at last follow up (117 months) and of the right knee (b) at last follow up (128 months).

Postoperative knee radiographs in anteroposterior and lateral views were performed at 1, 3 and 6 months following surgery and subsequently repeated annually to monitor implant positioning, joint alignment and any potential complications over time (Figure 3a,b).

Radiographic assessment included measurement of the hip‐knee‐ankle (HKA) angle on long‐leg standing anteroposterior radiographs to evaluate coronal limb alignment postoperatively. Additionally, standard anteroposterior and lateral radiographs were analysed for the presence and progression of radiolucent lines, with specific attention to tibial and femoral component zones.

### Statistical analysis

An independent statistician performed the statistical analysis. Categorical data were summarised using frequency counts and percentages, while continuous variables were presented as the arithmetic mean accompanied by the standard deviation (SD).

### Ethical considerations

This study adhered to the principles outlined in the Declaration of Helsinki and complied with Health Insurance Portability and Accountability Act (HIPAA) regulations. Institutional Review Board (IRB) approval was obtained from the Local Ethical Committee (approval no. 2015001968). Written informed consent was obtained from all patients for the surgical procedure and the use of anonymized data for research purposes.

## RESULTS

The mean surgical duration was 98.61 ± 18.28 minutes for single TKAs and 202 ± 29.28 minutes for bilateral procedures. Spinal anaesthesia was administered in 93.1% of cases, while 6.9% received general anaesthesia. The mean follow‐up period was 11.1 years (133 months ± 7.54).

### Patient‐reported outcomes

The average FJS‐12 at final follow‐up was 69.52 ± 12.21, indicating a high degree of joint awareness reduction. The mean final WOMAC score was 25.29 ± 16.97, reflecting satisfactory pain control and functional capacity. Similarly, the OKS reached a mean value of 38.63 ± 7.99, suggesting favourable clinical outcomes (Table 2).

**Table 2:** Outcomes data.

Clinical outcome	Average (pts)	SD
FJS‐12	69.52	12.21
OKS	25.29	16.97
WOMAC	38.63	7.99

Abbreviations: FJS‐12, Forgotten Joint Score‐12; OKS, Oxford Knee Score; SD, standard deviation; WOMAC, Western Ontario and McMaster Universities Arthritis Index.

### Radiographic assessment

All the implants resulted well positioned according to the angles described by Ewald and the average HKA angle was 179° ± 2.1°, when the mean pre‐op HKA angle was 175 ± 5.5°.

Serial imaging confirmed accurate implant positioning and stable limb alignment throughout the follow‐up period. Radiolucent lines suggestive of aseptic loosening were observed in two cases, both of which subsequently required revision surgery. No cases of component malalignment were identified on follow‐up radiographs.

At the radiological evaluation, performed according to the Ewald classification, except for the revision cases for aseptic loosening, in the other cases there were no progressive significant radiolucent lines. Four patients demonstrated non‐significant (<2 mm) and non‐progressive radiolucent lines in the femur (Zones 3 and 4) and nine patients had small non‐progressive radiolucent lines around the tibial component (Zones 1–2 and 4).

The mean *α* angle (femoral component valgus) was 92.7° ± 2.8, the *β* angle (tibial component varus/valgus) was 88.6° ± 1.2, and the *γ* angle (femoral component flexion) averaged 2.9° ± 2.3. The *δ* angle (tibial component posterior slope) was 85.9° ± 2.4, and the mean HKA angle was 179.1° ± 2.1 (Table 3).

**Table 3:** Radiographic data according to Ewald et al. [9].

	Alfa (*α*)	Beta (*β*)	Gamma (*γ*)	Delta (*δ*)	HKA
Average angle (SD)	92.7° (2.8)	88.6° (1.2)	2.9° (2.3)	85.9° (2.4)	179.1° (2.1)

Abbreviation: SD, standard deviation.

### Complications and revision rates

During the follow‐up period, five knees (4.31%) required revision surgery. The most common causes were aseptic loosening (*n* = 2, 1.72%) and persistent painful prosthesis without identifiable mechanical cause (*n* = 2, 1.72%). One case (0.86%) was revised for patellar clunk syndrome. The mean time to revision was 50.3 ± 55.7 months (Table 4).

**Table 4:** Time and causes of revision.

	Number	%
Total	5	4.31
Aseptic loosening	2	1.72
Persistent painful prosthesis	2	1.72
Patellar clunk syndrome	1	0.86
	Mean	SD
Time to revision (months)	50.33	55.72

### Implant survivorship

**Figure 4:**
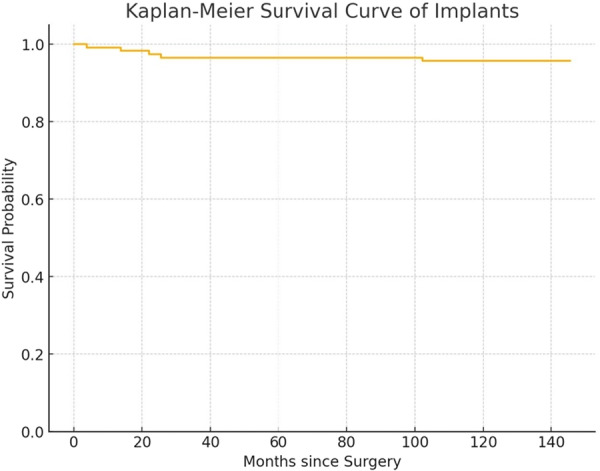
Kaplan–Meier survival curve of implants.

Two patients (4.3%) underwent revision surgery: one due to aseptic loosening and one for persistent painful prosthesis. Kaplan–Meier survival analysis, using revision as the endpoint, demonstrated an implant survival rate of 95.7% ±1.9% SD at a mean follow‐up of 11.1 years (Figure 4).

## DISCUSSION

The most important finding of the present study is that it demonstrates excellent long‐term clinical and radiological outcomes of the Persona Knee Posterior Stabilized System, with 95.7% implant survival rate at a mean follow‐up of 11.1 years, high patient‐reported outcome scores, and a low complication rate.

At the best of our knowledge, this study demonstrates the results of the anatomic design of the Persona System at longest follow‐up in literature.

TKA has undergone continuous advancements aimed at improving patient satisfaction and functional outcomes [26]. In this pursuit, factors such as implant alignment, level of insert constraint, surgical techniques, and emerging technologies—such as robotic‐assisted surgery—have received considerable attention [15, 18, 30, 35]. However, prosthetic design itself remains a fundamental determinant of clinical success.

Our findings indicate that the anatomic design used in this cohort led to excellent functional outcomes, while the revision rate remained low, with only five cases requiring revision surgery. In particular, we focused on analysing the FJS‐12 as our aim was to evaluate whether an anatomic prosthetic design could, over a long follow‐up period, enable patients to perceive their knee as natural to the point of “forgetting” they had undergone TKA. In our cohort, the average FJS‐12 at a mean follow‐up of 11.1 years was 69.52 (SD 12.21), indicating a high level of joint awareness reduction and long‐term patient satisfaction. In comparison, Irmola et al. reported FJS values of 25, 54 and 53 at 3, 12 and 24 months respectively for the Persona group in a randomised controlled trial involving three different implant designs. While FJS values appeared to plateau at around 54 after 1 year in their study, our higher long‐term score suggests that patients may continue to experience increasing levels of joint integration and reduced awareness beyond the 2‐year mark [13]. In comparison, Mortensen et al. reported a mean FJS‐12 of 52.7 at 2‐year follow‐up for the Persona system, while our cohort reached a higher mean FJS‐12 of 69.52 (SD 12.21) at a longer follow‐up of 11.1 years, suggesting sustained and potentially improved joint awareness reduction over time [24]. Studies analysing standard TKA designs often report lower FJS‐12 scores, likely due to residual joint awareness associated with less natural knee kinematics.

Furthermore, our study demonstrated excellent results in terms of the OKS, with a mean final score of 38.63 (SD 7.99), reflecting significant improvements in pain relief and functional outcomes. These findings align with and extend the results of previous studies evaluating the Persona knee system, even though the investigated cohorts were followed up at shorter period of time. Mathijssen et al. presented a prospective observational study assessing the Persona knee implant at 2 years postoperatively reporting an OKS increase from 22.1 to 41.8, indicating substantial functional improvement [22]. Similarly, Mahmood et al. evaluated a large cohort of 749 knees with a mean follow‐up of 5.35 years and found a mean OKS improvement of 20.7 points, with 94.9% of patients reporting satisfaction [20]. Our long‐term data at a minimum follow‐up of 10 years provide further evidence that this anatomic design continues to deliver sustained functional benefits over time.

Regarding implant survivorship, the Kaplan–Meier analysis demonstrated a survival rate of 95.7% at 11.1 years of follow‐up. In this study we observed a revision rate of 4.3% is within an acceptable range for primary TKA, with aseptic loosening and persistent pain being the main causes for revision. One case of revision was due to patellar clunk syndrome, a known complication associated with posterior‐stabilized designs. Despite this causes of revision, the overall rate of complications remained very low, showing the long‐term durability of this type of implant. Sung et al. reported in a prospective multicenter study an excellent 3‐year implant survival rate of 99.3% (95% confidence interval [CI]: 98.4%–99.7%) with only five revisions over 5 years, demonstrating the durability of the Persona Knee system with cemented, posterior‐stabilized components [16]. De Villeneuve et al. reported on a cohort of 237 TKAs a 5‐year implant survival rate of 98.72% (95% CI: 0.95–1.00) for the same morphometric posterior‐stabilized TKA, highlighting its solid mid‐term performance and low revision rate [4]. Dauder Gallego et al. reported a cumulative implant‐related revision rate of 3.3% at a mean follow‐up of 5.9 years for the Persona system, indicating good mid‐term durability [8].

However, their shorter follow‐up limits direct comparisons on long‐term survivorship and late complications. Our extended follow‐up strengthens the analysis, providing valuable insights into the long‐term performance of anatomic knee designs amid evolving surgical techniques and alignment strategies.

The complication rate in our study was very low, with only five revision procedures out of 116 knees (4.3%) over a long follow‐up time. The primary reasons for revision were aseptic loosening (1.7%), persistent painful prosthesis (1.7%) and patellar clunk syndrome (0.9%). These findings are consistent with previously published literature on total knee arthroplasty using anatomic implants, which report similar or slightly higher revision rates over comparable follow‐up periods [7].

Several factors may have contributed to the favourable outcomes observed in this study. The use of anatomic components tailored to individual patient morphology likely played a significant role in achieving better kinematic balance and joint stability. This aligns with the findings of Benazzo et al., who demonstrated that the use of a personalised TKA design, such as the Persona knee system, resulted in excellent radiological outcomes. The study showed optimal restoration of key anatomical parameters, including posterior condylar offset and tibial coverage. These results suggest that the anatomic nature of the prosthesis, combined with a reproducible surgical technique, facilitates precise implantation, ultimately contributing to improved joint function and stability [3].

Limitations for this study must be acknowledged. The retrospective nature of the study introduces intrinsic biases, including loss to follow‐up (33 patients and 2 bilateral TKAs), which may affect the generalisability of the findings. The study was conducted in a single centre and only with a Posterior Stabilized implant. No control group is present.

Then, while clinical and radiographic assessments were performed, more advanced imaging techniques (e.g., CT‐based kinematic analysis) could have provided further insights into implant positioning and wear patterns. Finally, while we observed no significant differences in outcomes based on sex or age, a larger sample size with subgroup analysis could help get more accurate results.

## CONCLUSIONS

This study demonstrates that an anatomic total knee arthroplasty design, such as the Persona Knee System, provides excellent long‐term clinical outcomes and sustained implant survivorship at a minimum follow‐up of 10 years.

The combination of precise surgical techniques, patient‐specific anatomic implant design, and meticulous soft tissue balancing likely contributed to these favourable outcomes.

Despite the study's retrospective nature and the loss to follow‐up of some patients, our findings support the continued use of anatomic knee prostheses to improve functional outcomes and patient satisfaction. Future research with larger cohorts and advanced imaging techniques may further refine implant design and surgical strategies, optimising the goal of achieving a truly 'forgotten knee' for TKA patients.

## AUTHOR CONTRIBUTIONS

Stefano Marco Paolo Rossi and Alice Montagna designed and with Francesco Benazzo were responsible for the study. Alice Montagna, Virginia Cinelli and Marina Marescalchi contributed to write the manuscript and Rudy Sangaletti finalised the statistical section, Marina Marescalchi, Luca Andriollo and Virginia Cinelli were responsible for the clinical x‐rays evaluation. Alice Montagna, Stefano Marco Paolo Rossi and Francesco Benazzo supervised the study and revised the manuscript.

## CONFLICT OF INTEREST STATEMENT

Stefano Marco Paolo Rossi and Francesco Benazzo declare consulting contract with Zimmer Biomet, Francesco Benazzo also declares royalties from Zimmer Biomet all the other authors declare no conflicts of interest.

## ETHICS STATEMENT

The study was approved by the Ethical Committee of our institution (IRB approval No. 2015001968). All patients signed an informed consent for the surgical procedure and for publication of the data.

## Data Availability

Data are available in a separate repository and can be disclosed upon reasonable request.
